# An Untargeted Metabolomics Strategy to Identify Substrates of Known and Orphan *E. coli* Transporters

**DOI:** 10.3390/membranes14030070

**Published:** 2024-03-20

**Authors:** Mohammad S. Radi, Lachlan J. Munro, Daniela Rago, Douglas B. Kell

**Affiliations:** 1 Novo Nordisk Foundation Center for Biosustainability, Technical University of Denmark, Søltofts Plads, Building 220, 2800 Kogens Lyngby, Denmark; 2 Department of Biochemistry, Cell and Systems Biology, Institute of Systems, Molecular and Integrative Biology, University of Liverpool, Crown St, Liverpool L69 7ZB, UK

**Keywords:** untargeted metabolomics, human serum, orphan transporters, *E. coli*, y-ome

## Abstract

Transport systems play a pivotal role in bacterial physiology and represent potential targets for medical and biotechnological applications. However, even in well-studied organisms like *Escherichia coli*, a notable proportion of transporters, exceeding as many as 30%, remain classified as orphans due to their lack of known substrates. This study leveraged high-resolution LC-MS-based untargeted metabolomics to identify candidate substrates for these orphan transporters. Human serum, including a diverse array of biologically relevant molecules, served as an unbiased source for substrate exposure. The analysis encompassed 26 paired transporter mutant contrasts (i.e., knockout vs. overexpression), compared with the wild type, revealing distinct patterns of substrate uptake and excretion across various mutants. The convergence of candidate substrates across mutant scenarios provided robust validation, shedding light on novel transporter-substrate relationships, including those involving *yeaV*, *hsrA*, *ydjE*, and *yddA*. Furthermore, several substrates were contingent upon the specific mutants employed. This investigation underscores the utility of untargeted metabolomics for substrate identification in the absence of prior knowledge and lays the groundwork for subsequent validation experiments, holding significant implications for both medical and biotechnological advancements.

## 1. Introduction

Transport systems, which selectively move substrates across cell membranes, constitute an important component of bacterial physiology. The ability to manipulate bacterial transporters both genetically and chemically has applications in both medicine (as a major component of antibiotic resistance due to evolved adaptations in efflux transporters [1,2,3,4]) and microbial cell factories (as a reliable way to increase product yield and tolerance of toxic products to increase efflux from the intracellular space into the extracellular medium [5,6,7,8,9,10,11,12]). Transporters are similarly required to maximize substrate uptake [13,14,15,16]. 

*Escherichia coli* is a model organism and has been intensively studied, with the full genome first published in 1997 [17]. Of the 4146 genes in the *E. coli* K-12 MG1655 chromosome, some 598 (around 15%) encode established or predicted membrane transporters [18]. Despite intensive research on *E. coli*, more than a quarter of these transporters (currently 160 to our best approximation) are considered y-genes or orphans (see also [19]), in that they have no assigned substrate. Even in the cases of those for which a substrate has been identified, it may be far from the best or main natural substrate (see, for example, [20]).

Finding substrates and elucidating the structure activity relationship of orphan bacterial transporters is a challenging proposition. Many nutrient importers have numerous transporters of different affinities and specificities (for example tryptophan and tyrosine can enter *E. coli* cells via several different transporters [21,22,23,24,25,26]) and given this redundancy, perturbation of a single transporter may not abolish import of the substrate, as well as the inevitability of inducing pleiotropic effects [27,28]. Furthermore, many efflux proteins export numerous xenobiotics, with substrates overlapping between transporters [29]. While there are techniques to allow measurement of transport directly on isolated transporters [30,31], this is only practically feasible when one has at least some idea of substrate selectivity. Given there are still many *E. coli* transporters with no assigned substrate (and that the annotation of transporters in other industrially relevant microorganisms is similarly lacking [32,33]), there is a need for a methodology that can in an unbiased way identify candidate substrates for orphan transporters.

Human serum is effectively a mix of many thousands of biologically relevant molecules [34,35], that provides a cheap and unbiased means of providing them to cells, in concentrations that plausibly reflect natural exposure [36]. Previously, incubation of human cells in serum followed by LC-MS has been used to identify substrates being imported and excreted into different cell lines using both low-resolution [20] and more recently high-resolution [37] methods. 

In the present study, we sought to apply these high-resolution methods to *E. coli* cells, utilizing single gene transporter overexpression cells derived from the ASKA collection [38] and single gene transporter knockouts from the KEIO collection [39]. Cells were incubated in human serum, and we measured changes in extracellular compound levels (the exo-metabolome [40] or metabolic footprint [41]) over a period of up to 30 min. We were able to detect a total of around 5000 compounds, many of which were identified with reasonably high confidence. We used automated analysis to screen time-course curves of wild type (WT) *E. coli*, with which we compared 26 transporter mutant pairs. Using this method, we observed clear differential uptake and excretion of diverse compounds. In addition, metabolite variations converged across mutant contrasts, as well as mutant-dependent associations, were systematically assessed for probing candidate substrates of putative transporters utilized in this study and aligned with their respective affiliation in the Transporter Classification Database [42]. 

## 2. Materials and Methods

### 2.1. Bacterial Strains and Cultures

The reference strain (the wild type) *E. coli* BW25113 utilized in this study was initially streaked on LB Agar. A selection of 26 transporters, as detailed in Table 1, was chosen for the experimental investigation. Each specific *E. coli* strain with a gene knockout corresponding to the selected transporters was obtained from the KEIO collection [39] and subsequently streaked onto LB Agar containing 25 µg/mL kanamycin for selection. Additionally, for each transporter gene of interest, independent overexpression vectors were obtained from the single gene overexpression (ASKA) collection [38]. Transformants were streaked on LB Agar supplemented with 25 µg/mL chloramphenicol for selection. For the preparation of overnight cultures, individual colonies were carefully picked and inoculated into 50 mL of LB medium, which was appropriately supplemented with the corresponding antibiotic. These cultures were incubated overnight at 37 °C with agitation at 300 rpm.

### 2.2. Serum Incubation Experiments

Overnight cultures were pelleted with centrifugation and then washed twice in 30 mL of glucose solution. OD was measured following the second resuspension in glucose to determine cell density. Cells were pelleted a final time and resuspended to a density of 6 × 10^9^ cells. A volume of 500 µL of the washed cells was dispensed in 96 deep well plates such that each recipient well contained 3 × 10^9^ cells. The plate was then centrifuged to pellet cells and the glucose solution removed. The runs were configured for both mutant contrasts alongside with the wild type *E. coli* BW25113 as a control in each run. For each cell type, three technical replicates were considered for each specified time point (0, 5, 15, and 30 min).

Cell pellets were resuspended in 200 µL of pre-warmed human serum (Pooled gender, purchased from BioIVT-West Sussex, Burgess Hill, United Kingdom) and incubated at 37 °C for the indicated time (30, 15, or 5 min). For the zero-time point, cells were resuspended in serum immediately before centrifugation. To control for breakdown or appearance of compounds over time within the serum, triplicate control wells of serum alone with no cells were included for all time points. Following incubation plates were centrifuged at 4000 rpm for 10 min at 4 °C and 150 µL of spent serum supernatant was transferred to a fresh plate and stored at −80 °C until purification. Pooled serum samples were prepared by mixing the remaining spent serum from all wells. 

### 2.3. Preparation for Metabolomics Analysis

Samples were thawed and 100 µL of each sample was mixed with 350 µL of acetonitrile. From this mixture 250 µL was added to an Ostro Protein Precipitation and Phospholipid Removal Plate (Waters, Taastrup, Denmark) and filtered with positive pressure. Filtered samples were dried in a vacuum centrifuge and re-suspended in 100 µL of LCMS grade water. 

### 2.4. LC-MS(MS) Analysis 

The LC-MS(MS) analysis was performed using a Vanquish Duo UHPLC binary system (Thermo Fisher Scientific, Waltham, MA, USA) coupled to an Orbitrap IDX Tribrid Mass Spectrometer (Thermo Fisher Scientific, Waltham, MA, USA). The chromatographic separation was achieved using a Waters ACQUITY BEH C18 (10 cm × 2.1 mm, 1.7 μm) column equipped with an ACQUITY BEH C18 guard column kept at 40 °C. The mobile phases consisted of MilliQ© water + 0.1% formic acid (A) and acetonitrile + 0.1% formic acid (B). The initial composition was 2%B, held for 0.8 min, followed by a linear gradient till 5% in 3.3 min, and afterward, 100%B was reached in 10 min and held for 1 min before going back to initial conditions. Re-equilibration time was 2.7 min. Flow rate was kept constant at 0.35 mL/min and injection volume was 1 µL. The MS measurements were performed in positive- and negative-heated electrospray ionization (HESI) mode with a voltage of 3500 and 2500 V, respectively, acquiring in full scan MS spectra in profiling mode using a resolution of 120,000 in the mass range of 70–1000 Da. The AcquireX workflow for automated generation of a background exclusion list from a blank sample was used during the data-dependent acquisition (DDA). The DDA acquisition settings were the following: automatic gain control (AGC) target value set at 4 × 10^5^ for the full MS and 5 × 10^4^ for the MS/MS spectral acquisition, the mass resolution was set to 120,000 for full scan MS and 60,000 for MS/MS events. Precursor ions were fragmented with stepped High-energy collision dissociation (HCD) using collision energies of 20, 40, and 60. The DDA spectra were acquired only on QC pooled samples. 

### 2.5. Data Preprocessing and Analysis

Data processing was performed using Compound Discoverer 3.1, essentially as described in [24]. Briefly, raw instrument data (.RAW) were imported to Compound Discoverer (CD version 3.1) and analyzed using the workflow described in Wright et al. [37]. Peak areas were exported, and a QC based LOESS signal correction was performed in R (version 4.2.3) as previously described [35] using the loess.as function of the fANCOVA package. The confidence level system utilized in the study aligns with established metabolomics reporting standards (see reference), ensuring a robust compound identification process. Criteria for confidence level assignment are based on matching scores exceeding 70 in mzVault and mzCloud databases (levels 1 and 2, respectively), a “Full match” in the Annotation Source (level 3), the presence of a molecular formula (level 4), and a confirmed molecular weight (level 5). 

Compounds being imported and exported were identified by automated analysis in R. Within this analysis package, basic univariate statistical analyses were conducted on log2 transformed data using a paired *t*-test. Volcano plots were then generated using these data, with a significance threshold of *p* < 0.05 and an absolute log2 fold change > 0.5 set to determine a significant alteration in compound abundance across time points. Following this, a Principal Components Analysis was employed to visualize trends in the differentially transported molecules.

Compounds were defined as being imported or exported if they: 1. could be fit to a linear regression with an adjusted R^2^ value greater than 0.7; 2. could be fit to an exponential regression with an adjusted R^2^ value greater than 0.7; or if 3. the compound showed greater than a 3-fold change over the first 15 min and a greater than 5-fold change over the 30 min of incubation. For identification of differences in transport for mutants, two criteria were explored. First, compounds which showed a 2-fold or higher change in peak area at the 5, 15, or 30 min time point compared to the WT strain, and without any overlap between the WT and mutant values at a given time point were selected. Second, strains where a significant difference between the WT and mutant values (*p* < 0.01) were also selected. Time-course graphs were generated to compare WT and each transporter mutant for all compounds that met these criteria and manually inspected for transport differences. These graphs underwent meticulous manual review to discern distinct transport discrepancies and deselect highly noisy data, and the finally selected molecules per a transporter mutant were exported in the form of tables. Comparisons were performed in R, and graphs were generated using ggplot2 package. All code used for data analysis is available along with the raw data at github.com.

## 3. Results and Discussion

### 3.1. Metabolite Profiling of Strains and Compound Identification

The experimental design involved the examination of transporter mutants alongside with the WT, and a serum-only control, with samples taken at 0, 5, 15, and 30 min. The selection of transporters, as detailed in Table 1, predominantly focused on transporters that have not been associated with specific substrates in previous research (i.e., uncharacterized, or putative), aiming to uncover potential new substrate-transporter relationships. Additionally, a selection of transporters with (at least some) known substrates was also included in the study. This dual approach aimed to confirm the method’s accuracy in replicating known or similar interactions while simultaneously investigating potential candidate substrates for uncharacterized transporters. 

For compound identification, MS/MS analysis was performed exclusively on pooled QC samples and processing was carried out simultaneously in Compound Discoverer (CD V3.1, see methods). The study adhered to the confidence level system of Schymanski et al. [43] with an overview provided in Table 2. In the positive Electrospray Ionisation mode (ESI+), 9406 compounds were detected, of which 3671 (39%) were unique (based on exact mass, retention time, and a maximum QC CV of 30%). MS/MS data were obtained for 5607 (60%) of these compounds, with 5305 (56%) showing the preferred ion. Identification confidence levels included 16 level 1 matches (0.2%) to reference standards, and 1363 level 5 identifications (14%) based on mass. In the negative Electrospray Ionisation mode (ESI-), 4092 compounds were identified, with 1632 (40%) unique and 3287 (80%) providing MS/MS data, including a higher proportion of level 1 identifications at 67 compounds (2%). A significant portion of the annotations are considered putative, with 75% in ESI+ and 62% in ESI- categorized as level 3 of identification. Compounds with confidence levels 1 through 3 were predominantly included, and their corresponding levels were indicated in figure legends in the following sections. Efforts are underway to develop more comprehensive in-house spectral libraries, which are expected to be integrated into subsequent analyses. The LC-MS/MS method demonstrates a high-capacity for identifying diverse metabolites reliably and accurately, benefiting from the synergistic use of both ESI+ and ESI- modes in metabolomic analysis.

### 3.2. The Differential Transport of Metabolites

To evaluate the differential uptake and excretion of serum components following incubation at different time points, a simple univariate analysis was performed using the data obtained at the start and the end of the incubation period (i.e., at time points 0 and 30 min). The WT cells and all transporter mutants utilized in this study showed apparent differences in the number and magnitude of metabolites being taken up or excreted, similarly to what has been observed previously with different eukaryotic cell lines [37]. Examples of volcano plots showing this differential analysis are shown in Supplementary Figure S1. In addition, principal component analysis (PCA) showed a clear difference in metabolite transport in response to transporter modulation and captured an apparent time-dependency in metabolite transport kinetics. Examples of PCA scores plots of some representative mutants are shown in Supplementary Figure S2.

**Figure 1:**
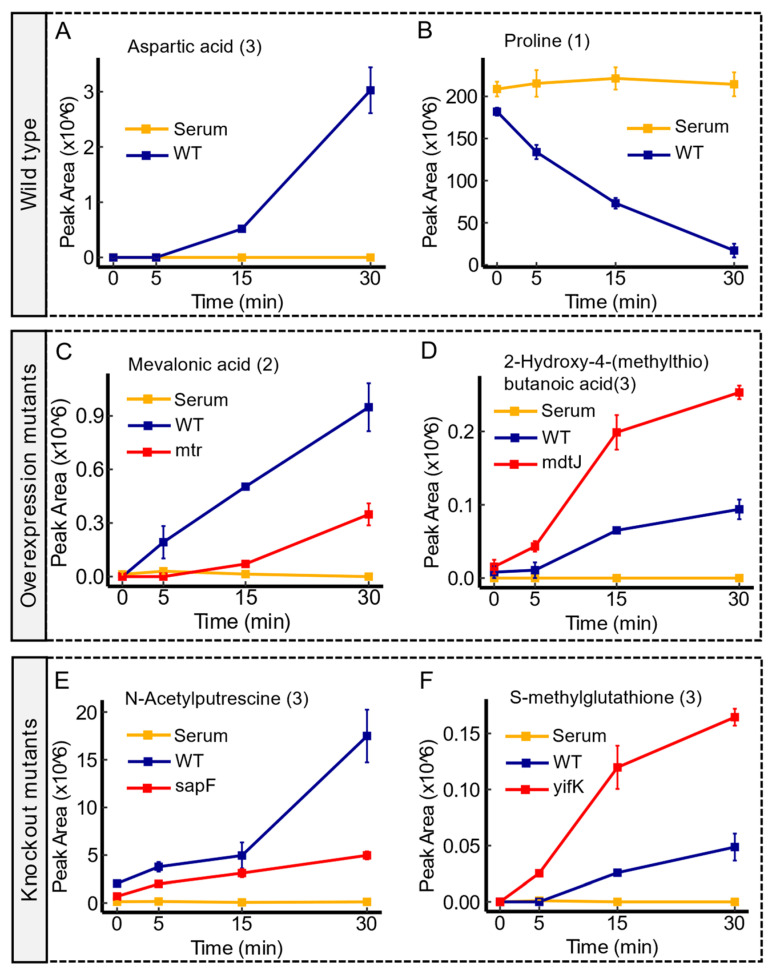
Examples of time-course analysis of transport dynamics in the wild type and transporter mutants. Panel (**A**,**B**) showcase an export of aspartic acid, and an import of proline, respectively, in the WT. Panel (**C**,**D**) outline the transport profiles of mevalonic acid and 2-Hydroxy-4-(methylthio)butanoic acid in mtr and mdtJ overexpression mutants, respectively. The dynamic alteration, characterized by an enhanced depletion (uptake) of mevalonic acid in mtr mutants compared to the wild type, suggests an increased uptake associated with the enhanced expression of mtr permease. However, the observed trend in peak area between the mdtJ mutant and the wild type suggests an enhanced export of 2-Hydroxy-4-(methylthio)butanoic acid under overexpression of mdtJ. Panel (**E**,**F**) delineate the transport profiles of N-acetylputrescine and S-methylglutathione in sapF and yifK knockout mutants, respectively. Compared to the wild type, the observed trend in sapF mutant shows an impaired export of N-acetylputrescine. Conversely, the trend of the peak area in yifK mutant versus the WT implies an impaired import function of S-methylglutathione. Serum controls are represented in yellow, WT in blue, and transporter mutants in red. Confidence levels for compound identification are indicated in brackets. Error bars denote standard errors, *n* = 3.

At the level of single molecules, several data filtration criteria were set to perform the initial evaluation of their transport profiles and interpret their potential transport direction based on the respective genetic perturbations (see methods for details). Briefly, time course data were examined to select molecules that demonstrated significant changes in concentration over time. Filtration criteria were applied to identify molecules that exhibited differential transport dynamics between the WT and each transporter mutant. Statistical analysis confirmed the significance of these differences. Eventually, time-course graphs were generated to visualize variations between WT and each transporter mutant for the selected molecules. Examples of time-course graphs illustrating the transport profiles of example metabolites are shown in Figure 1. The observed trends in peak area changes provide insights into the dynamics of metabolite uptake (depletion) or excretion (increase or export) over time. By comparing the trends in peak area changes of a molecule between the wild type cells and either the knockout mutant or the overexpression mutant, it is possible to infer a tentative direction of transport. Detailed findings on a broader array of putative transporter mutants are presented in the next section.

### 3.3. Probing Candidate Substrates of Putative Transporters

**Figure 2:**
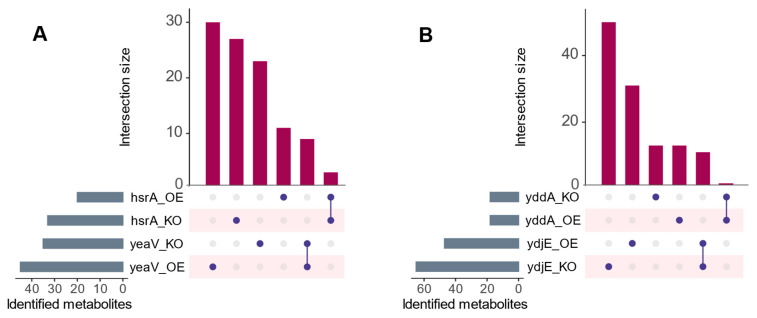
UpsetR [44] plot showing metabolites that are shared and unique across different examples of mutant pairs. (**A**) shows *hsrA* and *yeaV*, and (**B**) shows *yddA* and *ydjE* mutant pairs. Each horizontal bar represents a specific set of metabolites identified in each respective mutant, while the vertical bars show the size of intersections between mutant pairs (i.e., the number of metabolites that are common to both mutants being compared). Depicted is the possible convergence and divergence of metabolites that showed differential transport relevant to the parental strain. Despite the presence of converged instances between mutant contrasts, mutant-dependent metabolite variations were more prevalent. OE abbreviates overexpression mutants and KO abbreviates knockout mutants.

The intricate interplay between putative transporters and their cargo was explored based on a systematic comparison of metabolite dynamics in paired knockout and overexpression mutants (referred to as mutant contrasts). First, the convergence of metabolites between mutant contrasts was assessed to enhance the confidence in the selected candidate substrates. Additionally, the mutually interpreted transport directions of these candidates in mutant contrasts provided a dual validation approach that further reinforces the understanding of the transporter-substrate relationships. Secondly, mutant-dependent metabolite variations (i.e., diverged occurrences that were encountered where a range of metabolites exhibited unique associations with either the overexpression or knockout mutants) were also considered. The identified instances in this study demonstrated that the extent of this convergence or divergence varied across mutants. Examples of some pairs of mutants are shown in Figure 2 to illustrate this. Eventually, all findings were aligned with the respective transporter affiliation in the Transporter Classification Database [42]. In this context, a selection of representative putative transporters from various transporter families are provided as illustrative examples. Comprehensive data for all the tested transporters can be found in the Supplementary Material and Supplementary Data S1, which includes a set of fully tabulated data.

### 3.4. Candidate Substrates of yeaV

**Figure 3:**
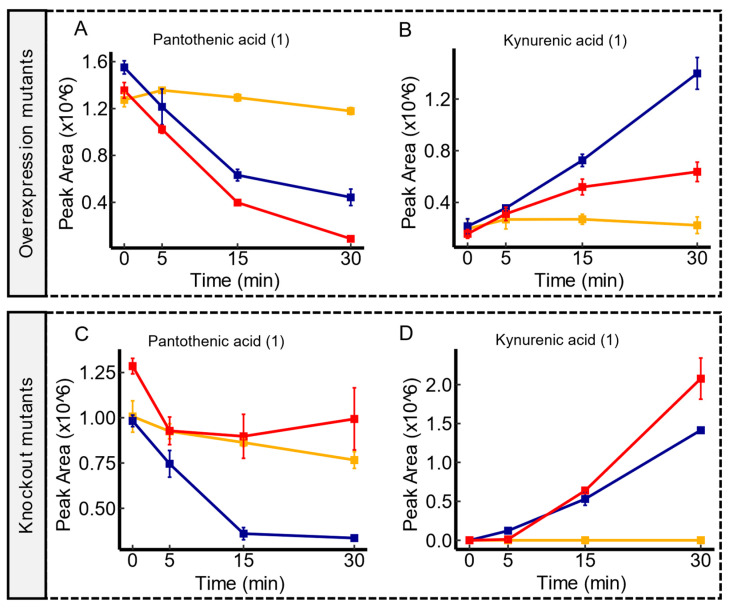
Temporal Transport Dynamics of pantothenic acid and kynurenic acid in *yeaV* mutant contrasts. Panel (**A**,**B**) illustrate a pronounced depletion of both metabolites in *yeaV* overexpression mutants in comparison to the WT. In contrast, the wild type displayed a more substantial depletion of both metabolites in comparison to *yeaV* knockout mutants as shown in panel (**C**,**D**). Serum controls are represented in yellow, WT in blue, and transporter mutants in red. Confidence levels for compound identification are indicated in brackets. Error bars denote standard errors, *n* = 3.

Within this dataset, *yeaV* emerged as a noteworthy putative transporter that showed associations with a diverse array of metabolite alterations in mutant contrasts. Examples of these metabolites are pantothenic acid and kynurenic acid. As shown in Figure 3, *yeaV* overexpression mutants displayed a greater reduction in the extracellular levels of both metabolites when compared to the WT, indicating an enhanced uptake activity for these molecules. Conversely, the WT exhibited a more substantial depletion of both metabolites over time compared to *yeaV* knockout mutants, suggesting an impaired uptake mechanism due to the loss of *yeaV* function. In addition, a similar metabolite shift suggested a decent uptake of ureidosuccinic acid, aspartic acid, and gallic acid (see Figure S3). Complementarily, there was a discernible depletion of several amino acids (or their acetylated forms), such as carnitine, serine, glutamic acid, N-acetyl-aspartic acid concomitant with *yeaV* overexpression (see Figure S4). In TCD, *yeaV* is listed as an uncharacterized putative member of the Betaine/Carnitine/Choline Transporter (BCCT) family [42]. Transport proteins within the BCCT family are recognized for their role in shuttling quaternary ammonium compounds, including betaine, carnitine, and choline, which are pivotal in cellular processes like osmoprotection, metabolism, and stress responses. The observations reported here are in line with the BCCT established functions and endorses a potential versatility of *yeaV* to transport a broad array of substrates. 

### 3.5. Candidate Substrates of hsrA

**Figure 4:**
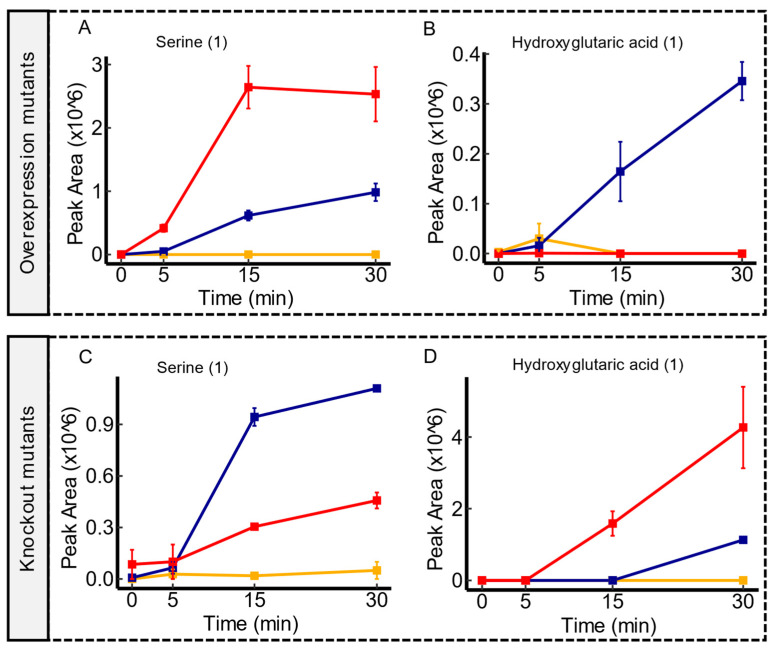
Time course illustrations of changes in serine and hydroxyglutaric acid in *hsrA* mutant comparisons. Compared to the WT, *hsrA* overexpression mutants displayed an enhanced extrusion of serine compared to the WT, as shown in panel (**A**). However, *hsrA* knockout mutants showed an impaired excretion of serine, as shown in panel (**C**). Panel (**B**) demonstrates a near depletion of hydroxyglutaric acid in *hsrA* overexpression mutants in comparison to the WT, while panel (**D**) displays the impaired uptake of this molecule in *hsrA* knockout mutants. Serum controls are represented in yellow, WT in blue, and transporter mutants in red. Confidence levels for compound identification are indicated in brackets. Error bars denote standard errors, *n* = 3.

In the investigation of *hsrA*, an uncharacterized member of the Drug:H+ Antiporter-2 (DHA2) Family of transporters [42], a diverse array of metabolite variations emerged. Given the pivotal role of antiporters in cellular metabolite transport, the two mutant scenarios employed in this study displayed an overlapping pattern in line with this function. As shown in Figure 4, *hsrA* overexpression mutant showed an enhanced excretion of serine compared to the WT, whereas *hsrA* knockout mutant showed a clear impairment in the excretion of this molecule in comparison to the WT. These findings suggest a potential involvement of *hsrA* in the export of serine under the experimental conditions used here. On the other hand, *hsrA* overexpression mutants exhibited a near depletion of extracellular hydroxyglutaric acid. In contrast, *hsrA* knockout mutant showed an impaired uptake of hydroxyglutaric acid compared to the parental strain. Similarly, consistent uptake trends were observed in both mutant scenarios for metabolites such as orotic acid, ureidosuccinic acid, and aspartic acid (Supplementary Data S1). Furthermore, there were additional independent instances in either knockout or overexpression mutants that suggest *hsrA*’s involvement in the transport of a variety of molecules including organic acids, phenolic compounds, and amino acids. Notably, among these molecules, methionine, and 5′-S-Methyl-5′-thioadenosine contain sulfur, aligning with previous findings of accumulating sulfur containing amino acids like homocysteine under *hsrA* overexpression. However, further investigations are required to fully elucidate these observations. Overall, these results align with the established functions of the DHA2 family and suggest a potential versatility of *hsrA* in transporting a wide range of substrates.

### 3.6. Candidate Substrates of ydjE

**Figure 5:**
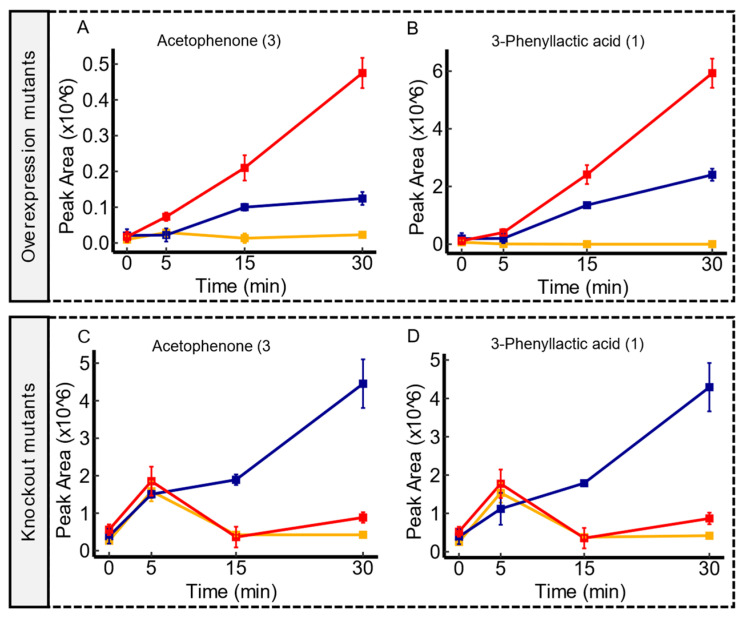
Time-course illustrations of changes in acetophenone and 3-phenyllactic acid by the *ydjE* mutant contrasts. Compared to the WT, *ydjE* overexpression mutants displayed an enhanced extrusion of acetophenone and 3-phenyllactic acid, as shown in panel (**A**,**B**), respectively. However, *ydjE* knockout mutants showed an impaired excretion of acetophenone and 3-phenyllactic acid in comparison to the WT, as shown in panel (**C**,**D**), respectively. Serum controls are represented in yellow, WT in blue, and transporter mutants in red. Confidence levels for compound identification are indicated in brackets. Error bars denote standard errors, *n* = 3.

The experiments conducted on *ydjE* transporter mutants demonstrated correlations with a broad spectrum of metabolites. Notably, two overlapping associations emerged in both mutant contrasts, strongly suggesting an export activity for both acetophenone and 3-phenyllactic acid. As illustrated in Figure 5, *ydjE* overexpression mutants demonstrated a consistent increase in the peak area throughout the incubation period, surpassing the WT, which implies (modulo pleiotropic effects), an enhanced export capacity, for these metabolites. Conversely, *ydjE* knockout mutants exhibited a clear impairment in the export of these metabolites when compared to the WT. Additionally, several associations were reported explicitly with either knockout mutants or overexpression mutants, showing the export of several molecules tentatively identified as amino acids (or their acetylated forms) and carboxylic acids (refer to Supplementary Data S1). Despite the affiliation of *ydjE* within the sugar porter (SP) family [42], a classification that was predicted based on sequence homology alone, the results showed here have revealed associations with metabolites that are not typically related to sugars. While the primary function of transporters in the SP family is sugar transport, it is not uncommon for transporters to exhibit promiscuity and transport other substrates as well in certain cellular contexts [45,46]. Nevertheless, the specific substrate preferences and regulatory mechanisms of *ydjE* are still unknown, and further research is needed to confirm the complete range of substrates transported by *ydjE* and the physiological significance of these transport activities in *E. coli*.

### 3.7. Candidate Substrates of yddA

**Figure 6:**
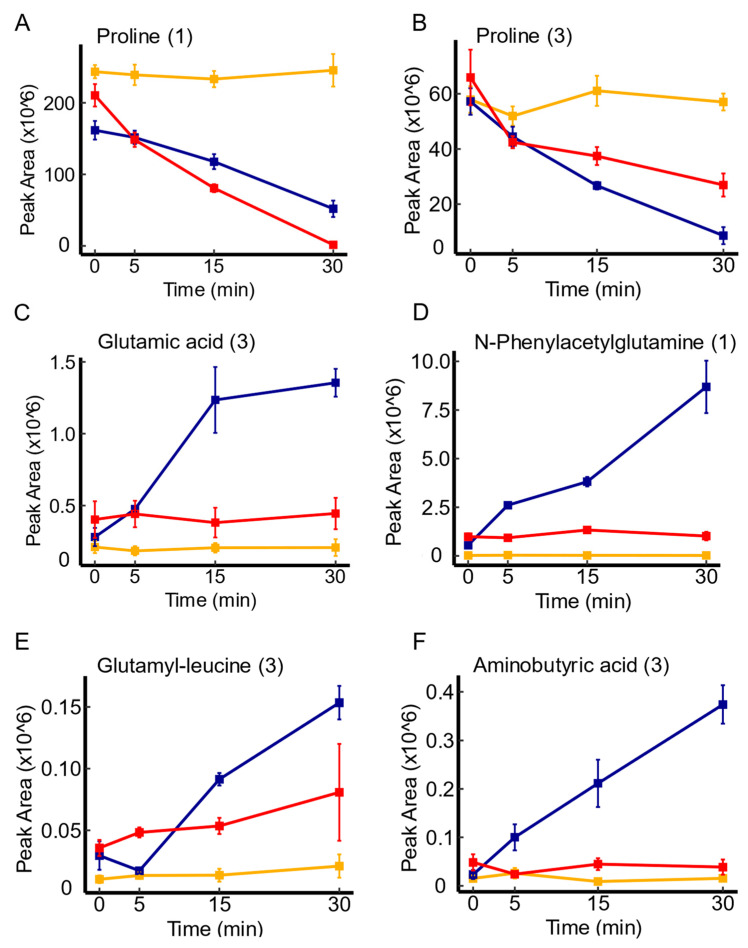
Time course illustrations of changes in several metabolites associated with *yddA* expression. Panel (**A**,**B**) represents the uptake pattern of proline in *yddA* overexpression and knockout mutants, respectively, in comparison to the WT. Panels (**C**–**F**) represent metabolite variations explicitly associated with *yddA* overexpression mutants, and depict a more efficient depletion patterns of the respective metabolites in comparison to the WT. Serum controls are represented in yellow, WT in blue, and transporter mutants in red. Confidence levels for compound identification are indicated in brackets. Error bars denote standard errors, *n* = 3.

In the investigation of *yddA*, a putative transporter of the ABC transporter family [42], mutant-dependent metabolite variations were more evident. Notably, *yddA* overexpression was linked to various metabolites, with amino acids and their derivatives being the most prevalent, including proline, glutamic acid, amino butyric acid, glutamyl-leucine, and phenylacetylglutamine. Figure 6 illustrate the enhanced depletion of these molecules over time in comparison to the WT. Of note, proline was the only molecule that showed convergence between mutant contrasts with an impaired uptake in *yddA* knockout mutants, highlighting the uptake pattern of this amino acid (see Figure 6A,B). In addition to amino acids, the analysis revealed similar uptake trends of organic acids (e.g., benzoic acid, ibotenic acid), aromatic compounds (e.g., 4-hydroxybenzaldehyde, coumarin), and essential primary metabolites like N(2)-succinyl-glutamic acid, in comparison to the WT. Previous studies have suggested that *yddA* is part of a three-gene cluster (*yddA*-yddB-ppqL) which may represent a 3-component iron-uptake system [47]. It has been found that overexpression of cloned *yddA* in the drug-supersensitive strain *E. coli* strain did not alter its resistance phenotype against many different drug and toxic compounds [28,29,48]. Moreover, previous investigations into fluorophore accumulation did not reveal substantial changes in mutants lacking *yddA* [28,29,48]. These prior findings, combined with our observations, support the potential role of *yddA* in the uptake of metabolites with biological significance such as amino acids. However, this would require further investigations. 

### 3.8. Remaining Transporters

The experiments performed here on 52 mutant strains, alongside with their parental strain, showed a relatively high capacity of the LC-MS method used here to operate at a reasonably high throughput. The cases represented earlier were illustrative examples; however, the remaining transporters and their respective candidate substrates are comprehensively listed in the Supplementary Data S1. The majority of the candidate substrates associated with the remaining transporters exhibited discernible metabolic alterations that were contingent upon the specific mutants employed. This observation underscores the intricate nature of transporter-substrate interactions, where the transport dynamics are influenced by specific genetic perturbations plus an unknown extent of pleiotropic effects. However, grouping of the candidate substrates based on their chemical properties or functional groups can provide insights on the preferred versus broader activity of a given putative membrane transporters. On the other hand, there was a moderate overlap between metabolites being imported or exported across mutants. Among these molecules were pantothenic acid, carnitine, adenine, uracil, organic acids, and some dipeptides. This convergence aligns with the notion of their essentiality to *E. coli* and their role in various cellular processes, and is likely best understood in the context of functional promiscuity of most membrane transporters [45,49,50].

## 4. Conclusions

Identifying substrates of microbial membrane transporters is of major relevance to medicine and industrial biotechnology. However, as with other genes in *E. coli*, more than 30% of the reported membrane transporter families remain insufficiently characterized or lack proper functional annotation [19,42]. Assigning a functional role to individual transporters based on the concept of “having a substrate, seeking a transporter” were solved using many suitable assays [5,28,30,31,51,52,53]. However, solving the inverse problem, “having a transporter, seeking substrate(s)”, is more challenging. In this study, a high-resolution untargeted metabolomics approach was harnessed to decipher candidate substrates of orphan transporters in *E. coli* illuminating dynamic uptake and excretion profiles exhibited by 26 transporter mutant pairs in comparison to their parental strain. Overall, the results presented here demonstrated: (1) the utility of untargeted metabolomics approach, coupled with the use of human serum, as a method to decipher candidate substrates of known and orphan transporters, obviating the need for prior knowledge. (2) The convergence of candidate substrates with a mutual metabolite shifts in mutant pairs provide a dual validation approach that further reinforces our findings. This convergence clearly underpinned the candidate substrates of many transporters such as *yeaV*, *hsrA*, *ydjE*, and *yddA*, all of which are novel assignments. (3) The use of intact *E. coli* cells provides a physiologically relevant environment for studying transporter-substrate interactions. This ensures that the observed transport activities are occurring in a structurally and functionally maintained integrity, while isolating the effect of specific transporters when deleted or overexpressed. 

Complex transporter networks and the tentative annotation of most of the identified compounds represent major challenges to the methodology introduced here. More specifically, the presence of functionally redundant transporters with broader substrates specificity may obscure the activity of the transport of interest. However, we have previously introduced an evolutionary selection-based approach that successfully addressed this challenge by combing both chemical and genetic perturbations to reveal such redundant mechanisms [46]. Despite the presence of many interesting cases where compounds’ annotations were tentative, the availability of computational tools capable of highly accurate molecular structure predictions from fragmentation data, as exemplified by Shirvastava et al. [54], offers a pathway for subsequent validation experiments. In addition, computational studies on transcriptomic data, such as weighted-gene correlation network analysis (WGCNA) and inference methods like independent component analysis (ICA), could infer a hypothesis about functions of putative transporters [19,55]. A synergetic combination of these computational inferences and the data obtained from this study can provide a good starting point to drive subsequent validation experiments in extended studies. Overall, the approach introduced in this study pave the way for the discovery of transporter functions and their biotechnological and medical applications.

## Figures and Tables

**Table 1:** A list of transporters selected for evaluation. Family and substrate information is derived from the Transporter Classification Database [42] (accessible at http://tcdb.org, accessed on 26 February 2024).

Transporter/TC# ^a^	Family	Previously Identified Substrates
yddG/2.A.7.17.2	Aromatic Amino Acid/Paraquat Exporter (ArAA/P-E)	Aromatic amino acids
Mtr/2.A.42.1.2	The Hydroxy/Aromatic Amino Acid Permease (HAAAP)	Tryptophan
ybbW/2.A.39.3.8	Nucleobase:Cation Symporter-1 (NCS1)	Allantoin
ynfM/2.A.1.36.1	Acriflavin-sensitivity (YnfM) Family	Arabinose
uraA/2.A.40.1.1	Nucleobase/Ascorbate Transporter (NAT) or Nucleobase:Cation Symporter-2 (NCS2)	Uracil
sapF/3.A.1.5.42	Peptide/Opine/Nickel Uptake Transporter (PepT)	Putrescine
yeeE(tsuA)/9.B.102.1.2	The YedE/YeeE (YeeE)	Thiosulfate
yifK/2.A.3.1.15	Amino Acid Transporter (AAT)	Serine and threonine
mdtJ/2.A.7.1.9	Small multidrug resistance (SMR)	Spermidine, SDS ^b^, deoxycholate
yojI/3.A.1.113.3	The Peptide-3 Exporter (Pep3E)	Microcin
ydfJ/2.A.1.6.9	Metabolite:H+ Symporter (MHS)	K^+^ *
ydhK/2.A.85.1.6	The Aromatic Acid Exporter (ArAE)	Uncharacterized
*yeaV*/2.A.15.2.3	The Betaine/Carnitine/Choline Transporter (BCCT) Family	Uncharacterized
yfdV/2.A.69.3.5	Auxin Efflux Carrier (AEC)	Uncharacterized
yfdC/1.A.16.4.1	Formate-Nitrite Transporter (FNT)	Uncharacterized
rarD/2.A.7.7.2	Chloramphenicol-Sensitivity Protein	Uncharacterized
*hsrA*/2.A.1.3.51	Drug:H+ Antiporter-2 (14 Spanner) (DHA2)	Uncharacterized
yfcJ/2.A.1.46.6	Uncharacterized Major Facilitator-5 (UMF5)	Uncharacterized
ydjX/9.B.27.1.1	Death Effector Domain A (DedA)	Uncharacterized
dcuD/2.A.61.1.2	The C4-dicarboxylate Uptake C (DcuC) Family	Uncharacterized
mdlA/3.A.1.106.13	Lipid Exporter (LipidE) Family	Uncharacterized
*ydjE*/2.A.1.1.92	Sugar Porter (SP)	Uncharacterized
*yddA*/3.A.1.203.11	The Peroxysomal Fatty Acyl CoA Transporter (P-FAT)	Uncharacterized
ygiS/3.A.1.5.41	The ATP-binding cassette superfamily	Uncharacterized
yiaV/8.A.1.1.4	The Membrane Fusion Protein (MFP) Family	Uncharacterized
yidE/2.A.81.1.5	Aspartate:Alanine Exchanger (AAEx) Family	Uncharacterized

^a^ Transporter Classification Database accession number, ^b^ sodium dodecyl sulfate, * Functions as an ion channel.

**Table 2:** Overview of LC-MS/MS results of serum QC samples obtained following preprocessing using CD3.1.

	ESI+	% All Compounds	ESI−	% All Compounds
Features Compounds Unique Compounds	531,376		135,776	
	9406		4092	
	3671	39%	1632	40%
MS2 No MS2	5607	60%	3287	80%
	3779	40%	805	20%
MS2 Preferred Ion	5305	56%	3213	79%
MS2 Other Ion	302	3%	74	2%
Level 1 (Match to reference standard) *	16	0.2%	67	2%
Level 2 (Probable structure, mzCloud > 70%) * Level 3 (Tentative Candidate, match to predicted composition) *	461	5%	70	2%
	7062	75%	2520	62%
Level 4 (Molecular formula) *	504	5%	251	6%
Level 5 (Mass) *	1363	14%	1184	29%

* Identification levels are based on criteria described by Schymanski et al. [43].

## Data Availability

The dataset supporting the results of this article is included in the article (and its Supplementary Files). Raw LC-MS data are available at github.com.

## Supplementary Materials

The following supporting information can be downloaded at: https://www.mdpi.com/article/10.3390/membranes14030070/s1, the dataset supporting the results of this article is included in the article (and its Supplementary files). Two files were attached separately: (1) Supplementary File containing all supplementary figures. (2) Supplementary Data S1 File: Spreadsheet containing all selected candidate substrates for each transporter mutant. Figure S1: Differential uptake and excretion of serum metabolites by the wild type (*E. coli*) and examples of different transporter mutants over a period of 30 min. Figure S2: PCA Scores plots of spent serum after incubation for 0 and 30 min with different transporter mutants. Figure S3: Temporal Transport Dynamics of uredosuccinic acid, aspartic acid, and gallic acid in *yeaV* mutant contrasts. Figure S4: Temporal Transport Dynamics of four molecules under the overexpression of *yeaV*.
