# Supplementary material for: An Untargeted Metabolomics Strategy to Identify Substrates of Known and Orphan E. coli Transporters
Source: Membranes (Basel). 2024 Mar 20;14(3):70. doi: 10.3390/membranes14030070 (PMC10971958; doi:10.3390/membranes14030070)
Supplement: Supplementary file 1 [file membranes-14-00070-s001.zip › Supplementary Material.pdf]

Supplementary Figures

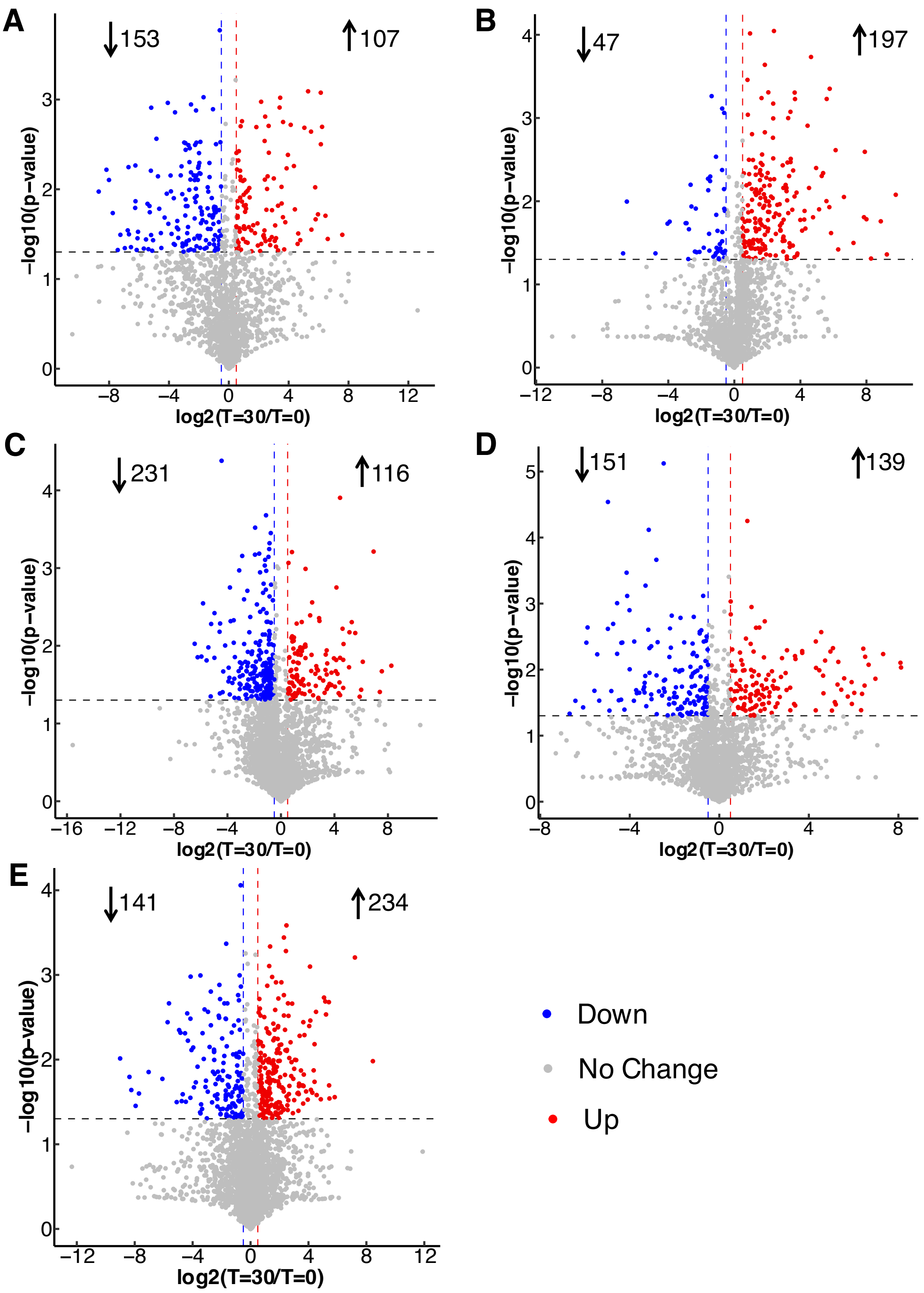

Supplementary Figure S1: Differential uptake and excretion of serum metabolites by the wild type (*E. coli*) and examples of different transporter mutants over a period of 30 minutes. Volcano plots illustrate the observed variations in the uptake and excretion of serum metabolites of A) the wild type *E. coli* BW25113, B) *ydjE* Knockout mutant, C) *yeaV* knockout mutant, D) *ydjE* overexpression mutant, and E) *yeaV* overexpression mutant. Univariate statistical analyses were carried out on log2 transformed data using a paired t-test. Volcano plots were generated based on log2 transformed data, with criteria set at $P < 0.05$ and an absolute log2 fold change > 0.5 to identify significant alterations in compound abundance between time points (0 and 30 minutes).

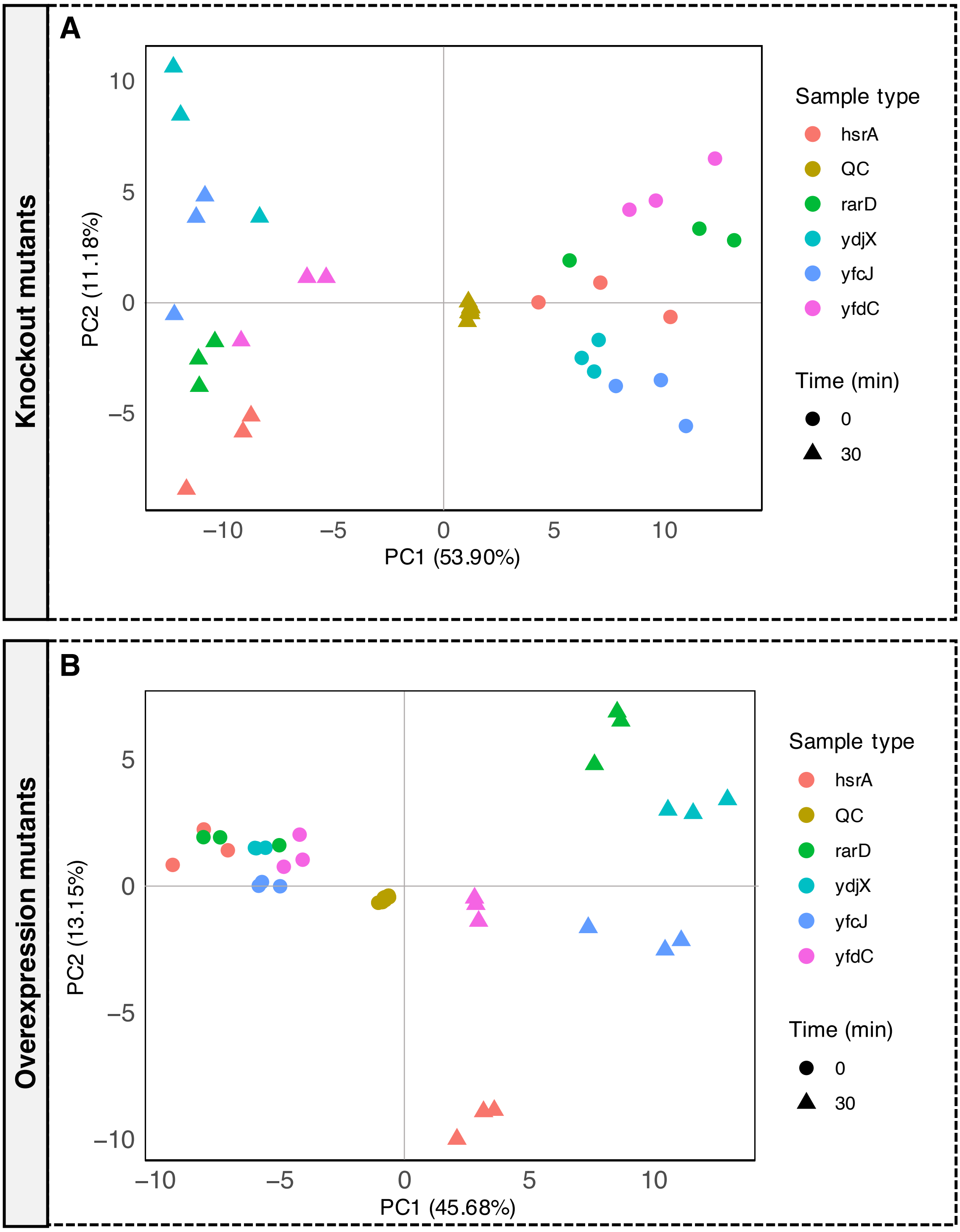

Supplementary Figure S2: PCA Scores plots of spent serum after incubation for 0 and 30 minutes with different transporter mutants. Shown is an example batch of A) knockout mutants, and B) overexpression mutants. Colors represents different sample types (mutants *vs* QC), and different shapes represents incubation times. Intermediate time points were removed for clarity.

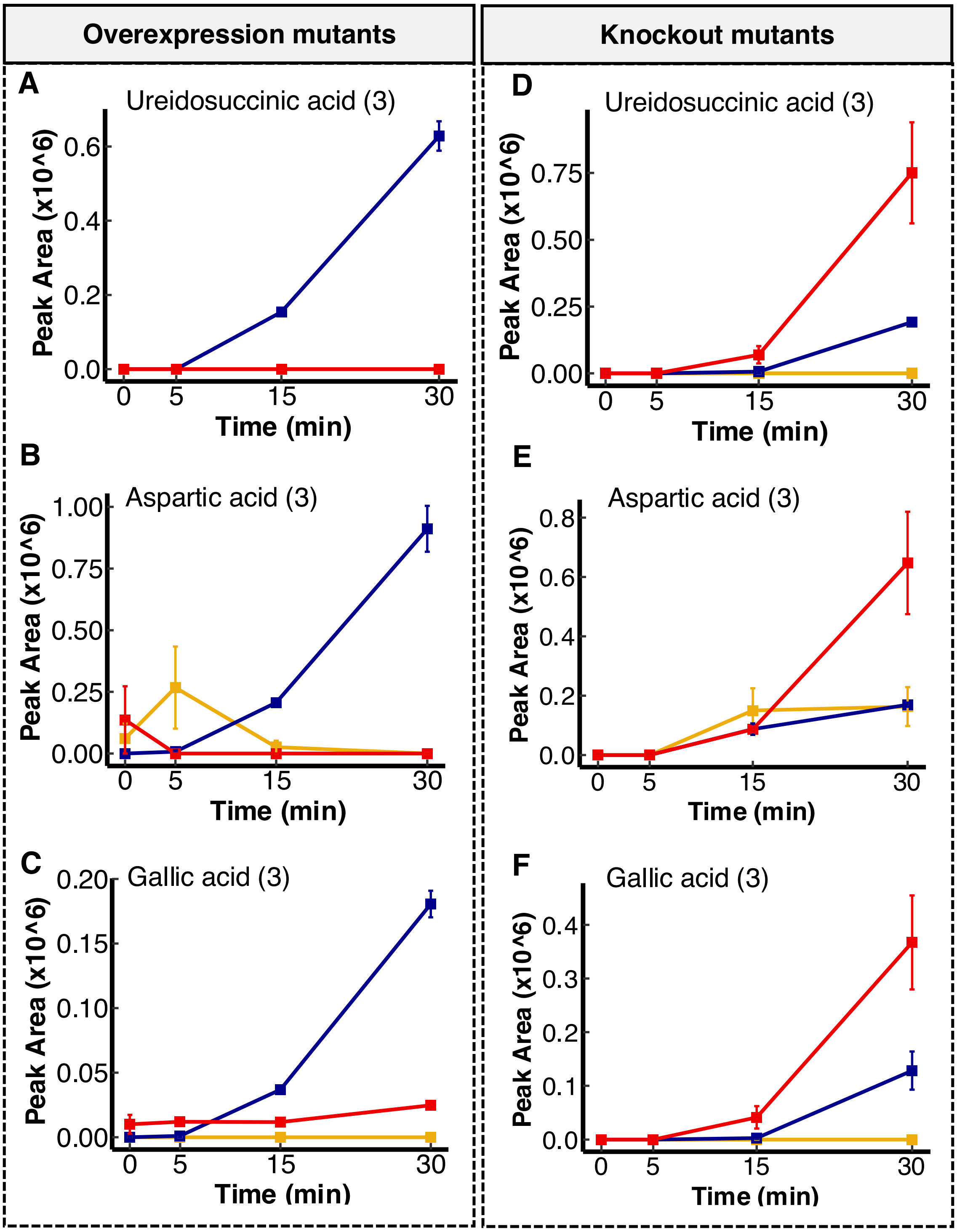

Supplementary Figure S3: Temporal Transport Dynamics of uredosuccinic acid, aspartic acid, and gallic acid in *yeaV* mutant contrasts. Compared to the wild type, *yeaV* overexpression mutants showed a significant depletion of these metabolites as shown in panel A, B, and C. In contrast, in *yeaV* knockout mutants, depletion of both metabolites was highly impaired in comparison to the wild type as shown in panel D, E, and F. Serum controls are represented in yellow, WT in blue, and transporter mutants in red. Confidence levels for compound identification are indicated in brackets. Error bars denote standard errors, n=3.

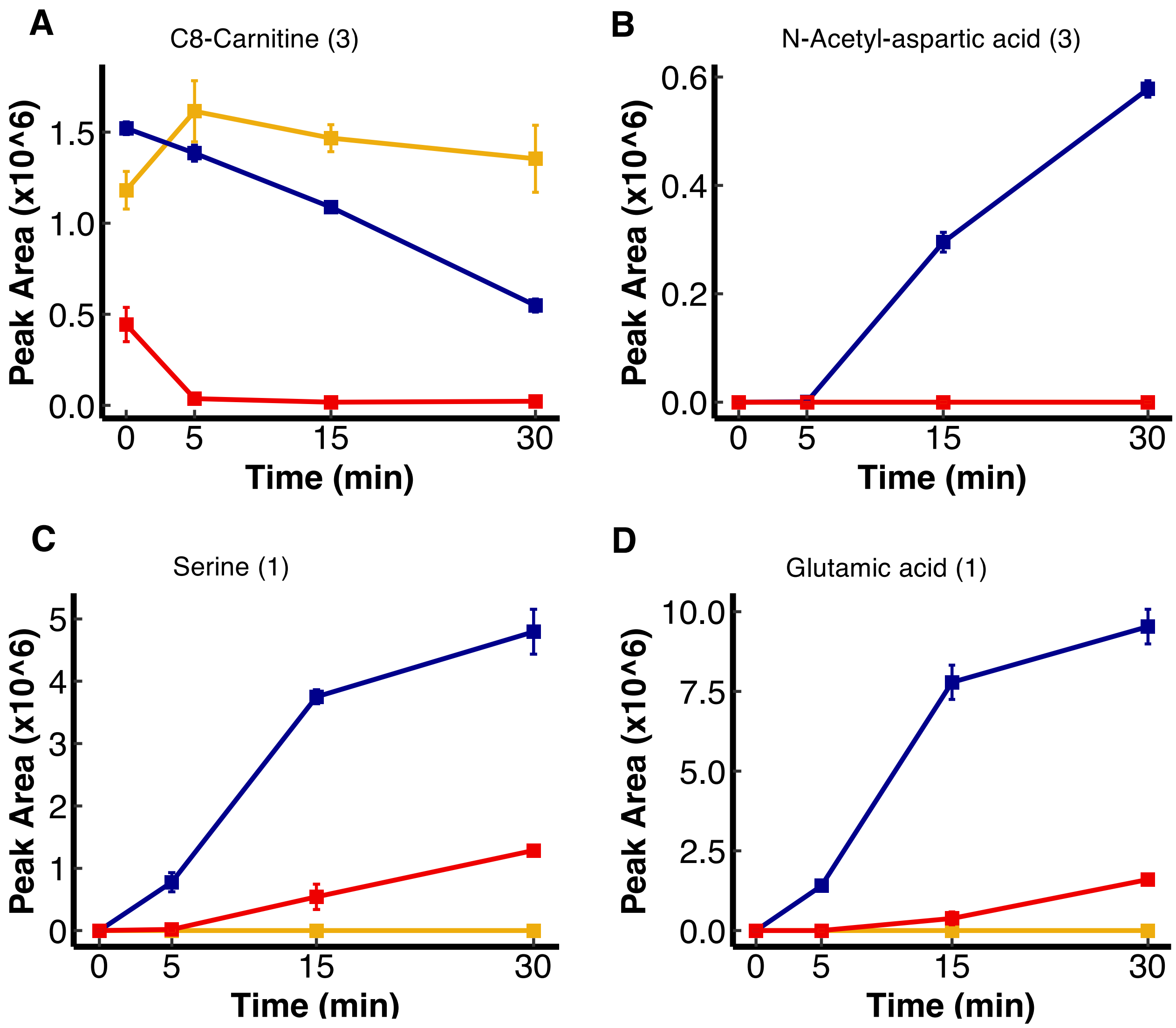

Supplementary Figure S4: Temporal Transport Dynamics of four molecules under the overexpression of *yeaV*. Compared to the wild type, *yeaV* overexpression mutants showed an enhanced uptake of A) C8-carnitine, B) N-acetyl-aspartice acid, C) serine, and D) glutamic acid. Serum controls are represented in yellow, WT in blue, and *yeaV* transporter mutants in red. Confidence levels for compound identification are indicated in brackets. Error bars denote standard errors, n=3.

Supplementary Data S1: Spreadsheet containing all selected candidate substrates for each transporter mutant and some examples of spectral reference matching.
